# Salter-Harris Type III Fracture-Dislocation of the Proximal Humerus

**DOI:** 10.7759/cureus.95816

**Published:** 2025-10-31

**Authors:** Andrew S Kucey, Khaled Skaik, Megan Cashin

**Affiliations:** 1 Orthopedic Surgery, Memorial University of Newfoundland, St. John's, CAN; 2 Medicine, McGill University, Montreal, CAN

**Keywords:** open reduction and internal fixation, orthopedic trauma, pediatric orthopedics, proximal humerus fracture, salter-harris type 3

## Abstract

Pediatric proximal humerus fractures are an uncommon injury; fracture-dislocations are even more rare. These are most often Salter-Harris (SH) type I or II injuries, and they rarely require operative intervention. This report details the management of an SH III fracture-dislocation in a 10-year-old. The incarcerated articular fragment necessitated an open reduction and was stabilized with percutaneous Kirschner wires. At the one-year follow-up, the patient has regained a full range of motion, and there are no signs of avascular necrosis. The current study adds to the paucity of literature on the management and outcomes of an exceedingly rare injury.

## Introduction

Proximal humerus fractures represent around 2% of all pediatric fractures, are seen more frequently in females, and occur at an average age of 9.5 years old [1,2]. Pediatric proximal humerus fractures are most commonly Salter-Harris (SH) type II in adolescents, whereas type I occurs less frequently across all ages before growth plate closure, with types III and IV being exceedingly rare [3]. SH injuries are classified by the fracture line in relation to the physis, with grading from I to V. Patterns include transphyseal (I), metaphyseal extension (II), epiphyseal extension (III), both epiphyseal and metaphyseal extension (IV), and physeal crush injury (V) [4]. The vast majority of these injuries are managed non-operatively due to the remodeling potential of the pediatric proximal humerus.

Here, we present the case of a 10-year-old boy who required operative management due to a rare SH III fracture with resultant anterior-inferior dislocation of the humeral articular surface.

## Case presentation

**Figure 1:**
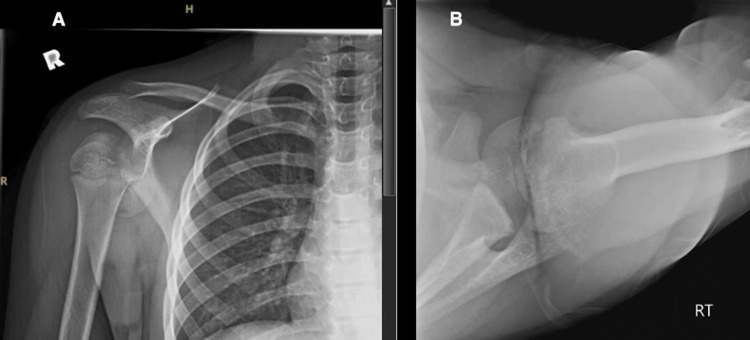
(A) Anteroposterior (AP) and (B) Velpeau radiographs showing an SH III fracture-dislocation of the right shoulder.

**Figure 2:**
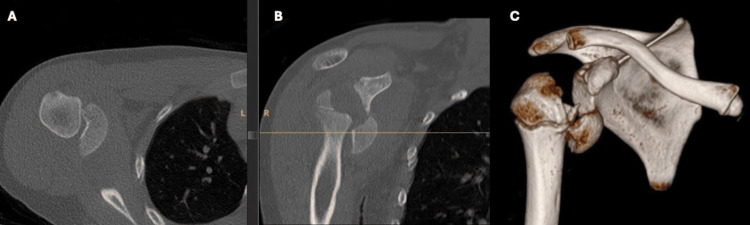
(A) Selected axial and (B) coronal computed tomography images of the SH III injury with (C) 3D reconstruction.

The patient was referred to the orthopedics team after he flipped over his handlebars while on a bicycle. Radiographs in the emergency department showed an SH III proximal humerus fracture with dislocation of the epiphyseal fracture fragment (Figure 1). Closed reduction in the emergency department, with the patient under conscious sedation, was unsuccessful. A computed tomography scan was completed, and urgent operative treatment was arranged to minimize the risk of avascular necrosis (Figure 2).

**Figure 3:**
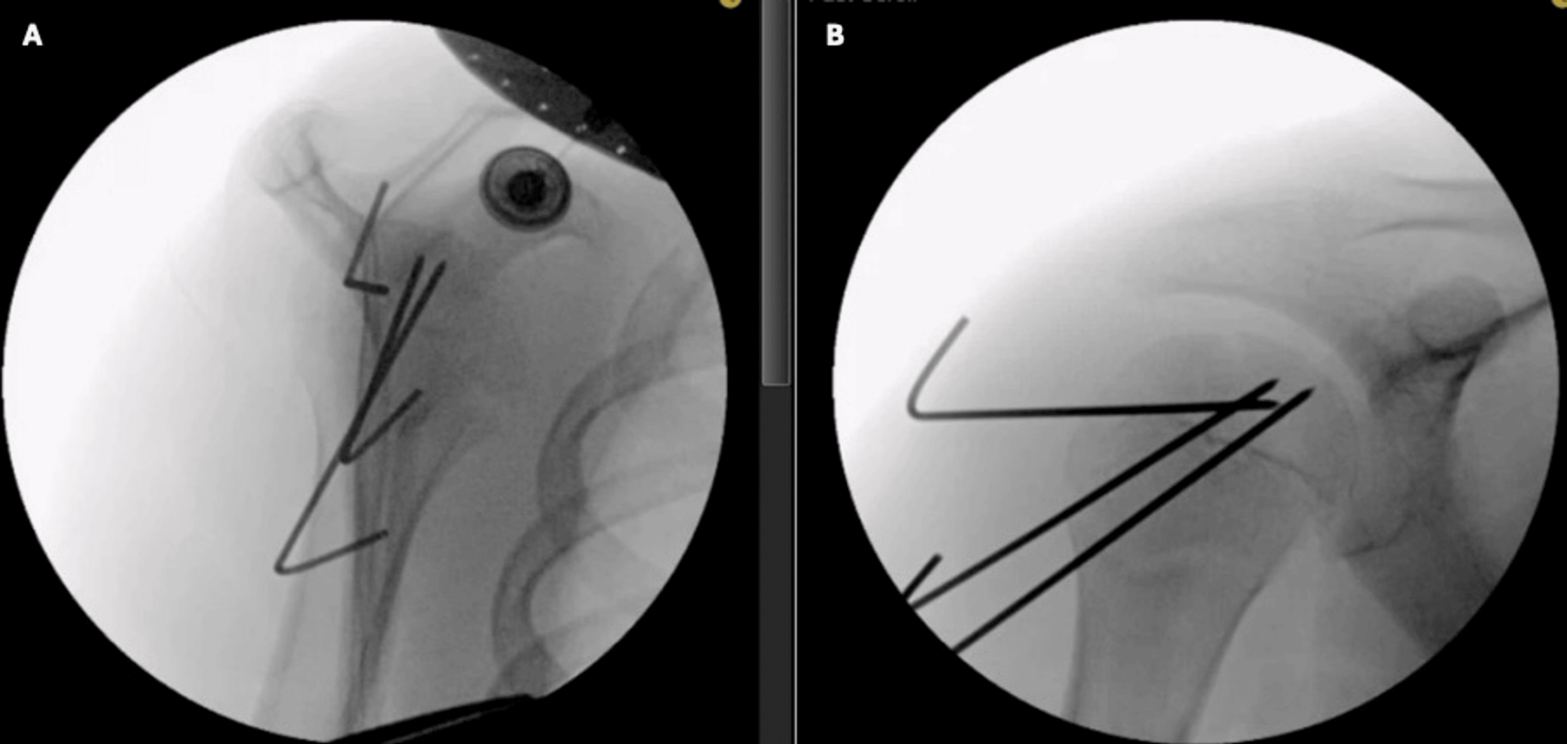
Intraoperative fluoroscopic images demonstrating a stable reduction of the SH III fracture and fixation with three smooth 1.6 mm Kirschner wires in the (A) lateral and (B) anteroposterior (AP) planes.

Once in the operating theater, closed reduction of the epiphyseal fragment was attempted with inline traction in addition to laterally directed pressure over the articular fragment via the axilla. The closed reduction was unsuccessful after two attempts, so an open reduction was performed via a deltopectoral approach. The joint space was accessed through the rotator interval. The articular surface was palpable in the infraglenoid pouch. A Cobb was passed underneath, and in conjunction with manual manipulation, the fragment was freed and reduced onto the proximal humerus. The reduction was stabilized with direct manual pressure while fixation was completed percutaneously with three smooth 1.6 mm Kirschner wires (Figure 3). The shoulder was ranged to ensure that a stable reduction was achieved.

**Figure 4:**
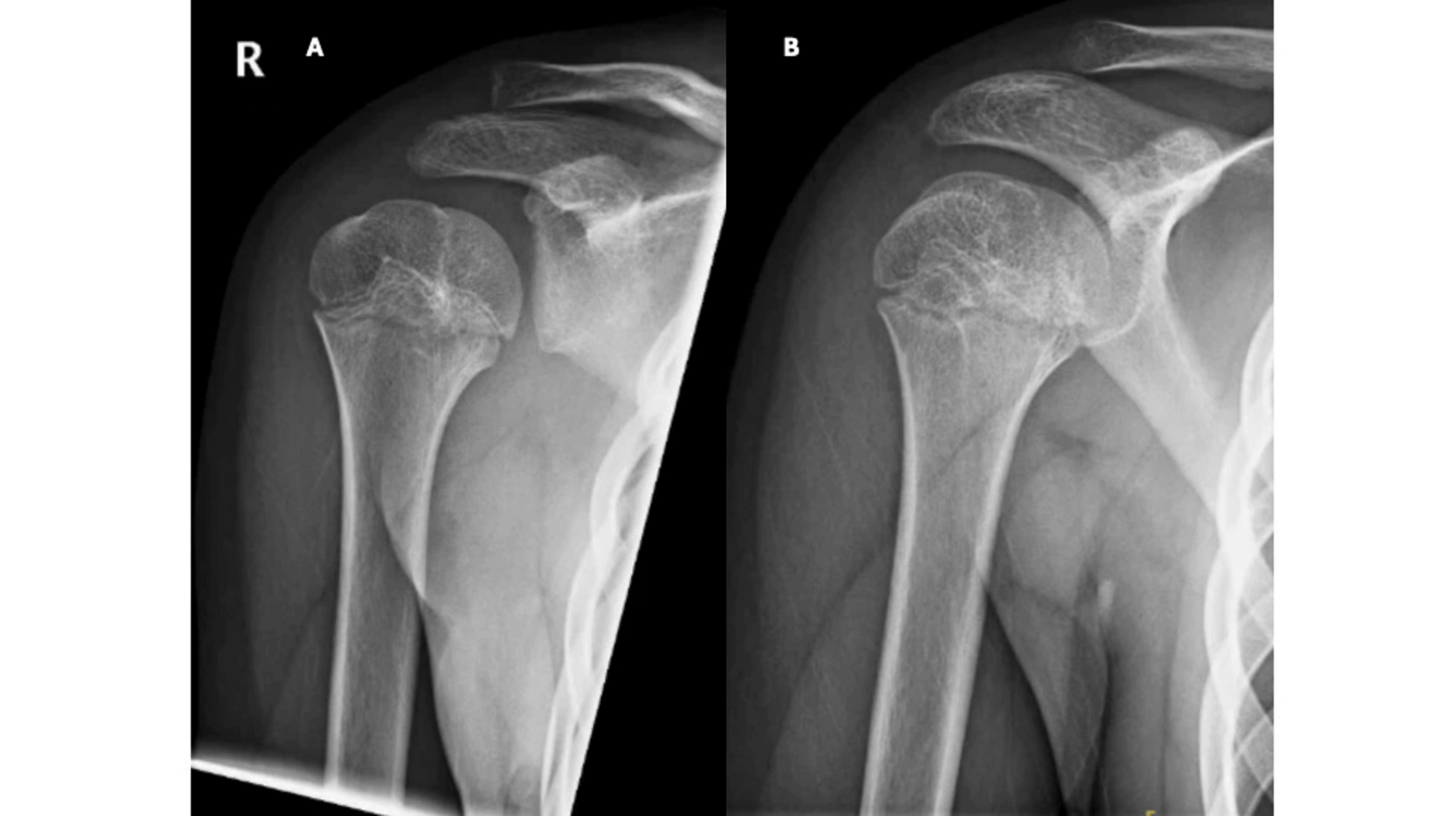
Follow-up radiographs showing a united fracture with no signs of avascular necrosis at 12 months. (A) Grashey and (B) anteroposterior (AP) views are shown.

Postoperatively, the patient was immobilized in a sling, and the pins were dressed in povidone-iodine-soaked sponges with gauze, abdominal pads, and Hypafix tape over them. The pins were removed at four weeks. He progressed to pendulum and then unrestricted range of motion (ROM) at his four- and eight-week follow-ups. The fracture united without complication on interval radiographs. At six months, the patient had unrestricted ROM, was pain-free, and had returned to full physical activities. At 12 months, there were no signs of avascular necrosis (Figure 4). He will continue to be monitored for the potential risk of growth arrest and avascular necrosis.

## Discussion

Traumatic proximal humerus fractures combined with glenohumeral dislocations are uncommon in the pediatric population. Most cases can be managed with closed reduction and a sling, while only a small number require surgical intervention [5]. Surgery is typically reserved for open fractures, severely displaced fractures, cases with neurovascular compromise, or irreducible fractures. Fracture-dislocations most commonly present as an SH type I or II [6,7]. Urgent intervention is warranted when there is a disruption of the blood supply to the epiphyseal segment. The risk factors for humeral head ischemia are classically described by Hertel et al., and they emphasized continuity of the posteromedial metaphysis plus the medial calcar hinge to preserve residual perfusion by the posterior humeral circumflex artery [8].

Many studies report SH types I and II, which were managed by a variety of closed or open reductions depending on the severity of the dislocation [9-11]. One of the earliest reported cases of pediatric shoulder fracture-dislocations was described by Nicastro and Adair in 1982 [10]. They reported on a 32-month-old child who presented with an SH type I fracture of the proximal humerus accompanied by an ipsilateral anterior shoulder dislocation. Treatment involved open reduction and fixation using a Kirschner wire. Winmoon et al. reported a case involving a two-year-old boy with an anterior shoulder dislocation and an SH type I proximal humerus fracture, which was treated by closed reduction alone [11].

In cases of SH type II fractures, Hong et al. detailed a nine-year-old boy who sustained a posterior shoulder dislocation along with an ipsilateral SH type II physeal fracture of the proximal humerus [12]. His injury was successfully managed using closed reduction and percutaneous pinning. Isik et al. reported a case involving a seven-year-old who sustained an SH type II fracture and glenohumeral dislocation of the proximal humerus following a fall from 1.5 meters, and that injury required open reduction [13].

Regarding SH III injuries, only six studies in the pediatric population have been published since 1986. These include four anterior dislocations, one posterior dislocation, and one that did not dislocate [14-17].

Wong-Chung and O'Brien reported on the non-dislocated SH III injury in a 10-year-old girl and successfully treated it with closed reduction alone [18]. Similarly, Cohn and Froimson reported on a 10-year-old boy with an anterior shoulder dislocation and concurrent SH III fracture. This was successfully treated with a closed reduction and did not require hardware [15]. However, in three other reported cases with anterior dislocations, open reduction was required [14,16,19]. These studies consistently found that the humeral head was locked against the anterior part of the glenoid, requiring a deltopectoral approach to reduce [10,12,15]. Multiple fixation methods have been utilized in these previous reports, including screws, Kirschner wires, and Steinmann pins.

Wang et al. reported a patient with avascular necrosis followed by later revascularization of the dislocated epiphyseal fragment [14]. They completed a bone scan postoperatively, which precluded causality from the initial injury or the surgical procedure due to its timing. At a two-year follow-up, there was no growth disturbance, a noteworthy outcome given the high likelihood of growth complications associated with SH type III injuries in this region [14]. While the injured humeral head shape appeared altered compared to the uninjured side, its overall sphericity and the integrity of the physis were preserved [14].

A similar result was observed in a posterior dislocation by Lee et al., where their patient experienced complications, including premature physeal closure and localized avascular necrosis of the humeral head [17]. Compared to other reported cases, the patient's older age, the severity of the initial trauma, and delayed treatment may have contributed to these outcomes. Despite both patients being asymptomatic with avascular necrosis, the authors noted the importance of long-term follow-up to identify potential future issues [14,17].

Similar to the previously discussed cases, we present a rare case of a 10-year-old with an SH type III proximal humeral fracture and anterior dislocation, in which the epiphyseal fragment could not be reduced using closed manipulation. This was limited to two attempts to avoid further insult to the physis and cartilage. It was then treated with urgent open reduction through a deltopectoral approach and 1.6 mm Kirschner wire fixation to minimize the risk of ischemia to the humeral head. To date, there are no signs of avascular necrosis or growth arrest.

## Conclusions

Based on a review of the literature and the current case, we recommend prioritizing the preservation of the articular surface when managing SH type III injuries of the proximal humerus. Undisplaced injuries can be successfully managed nonoperatively with immobilization, while displaced fractures and fracture-dislocations require anatomic reduction using either closed reduction or open reduction. Fixation strategies vary in the literature, but we demonstrate that Kirschner wire fixation is satisfactory for these injuries.
